# Clozapine-Induced Myocarditis: A Case Report of an Adolescent Boy with Intellectual Disability

**DOI:** 10.1155/2015/482375

**Published:** 2015-07-22

**Authors:** Lila Aboueid, Nitin Toteja

**Affiliations:** ^1^Department of Psychiatry, SUNY Downstate Hospital, 450 Clarkson Avenue, Brooklyn, NY 11203, USA; ^2^Department of Psychiatry, Kings County Hospital, 451 Clarkson Avenue, Brooklyn, NY 11203, USA

## Abstract

*Background*. Although known for its efficacy in treatment-resistant schizophrenia, the usage of clozapine has been limited due to concerns over potential adverse effects. Myocarditis, one potential fatal complication, can develop at any point during treatment but has been most commonly observed 2-3 weeks after clozapine initiation. *Objective*. A case of acute clozapine-induced myocarditis is described, highlighting the history, onset, and treatment course of presentation. There is a need to raise awareness of this potential complication, especially in the pediatric population. *Results*. 17-year-old Puerto Rican boy, with history of schizophrenia, disorganized type (treatment resistant), and intellectual disability, developed myocarditis on the thirteenth day following clozapine commencement. Initial presenting symptoms included tachycardia, lethargy, and vague gastrointestinal distress. Patient fully recovered after supportive medical care and clozapine discontinuation. *Conclusions*. Myocarditis is a known potential complication of clozapine initiation; however, due to its limited usage in the pediatric population, reported cases are limited. There is a need to establish evidence-based monitoring guidelines for clozapine usage, particularly in the pediatric population where the presentation may be atypical and clinical suspicion may be overlooked.

## 1. Introduction

Clozapine is a dibenzodiazepine derivative antipsychotic, unique for its high D1 and D4 dopaminergic receptor and 5-HT2a serotonergic receptor affinity [1]. Although known for its efficacy in treatment-resistant schizophrenia, its use has been limited due to potential adverse effects such as agranulocytosis [2], neutropenia, and myocarditis [3].

Kilian et al. [4] initially raised concerns of potentially fatal myocarditis and cardiomyopathy in physically healthy young adults (age 17–46) with schizophrenia treated on clozapine therapy. Their retrospective chart review of 8,000 patients showed that clozapine dosage at the time of diagnosis ranged from 100 mg to 725 mg daily. Of the 15 cases with myocarditis, symptoms appeared early in the course of treatment (median 15 days, range 3–21 days), consistent timing with one proposed causal mechanism of IgE-mediated hypersensitivity (type 1 allergic reaction). Although myocarditis can develop at any time during clozapine treatment, research has found that 83% of the 75 reviewed cases developed between days 14 and 21 [5].

Typically, clozapine causes an increase in heart rate (10–20 beats/minute) as a common side effect. However, clozapine-induced myocarditis often presents with nonspecific symptoms of illness like fever or respiratory, gastrointestinal, or urinary traction infection and progresses (1–5 days later) with elevated heart rate (20–30 beats/minute), troponin (>2 upper normal limit), c-reactive protein (CRP) >100 mg/L, and left ventricular impairment by echocardiogram [5]. Currently most guidelines and data are based on reported adult cases, with limited documentation (possibly due to limited utilization) in the pediatric population.

Here we present a pediatric case of a boy with intellectual disability who developed clozapine-induced myocarditis after initial signs of tachycardia, lethargy, and vague gastrointestinal distress.

## 2. Case Presentation

This is a 17-year-old Puerto Rican boy, with history of schizophrenia, disorganized type, and intellectual disability, admitted on September 27, 2014, for disorganized behavior and aggression towards peers and mother in the context of poor treatment adherence. Upon recent psychiatric inpatient discharge on September 16, 2014, patient reportedly stopped taking his prescription medications (quetiapine and guanfacine). On presentation, he was oddly related, was restless, was internally preoccupied, and reported having auditory hallucinations (unable to verbalize description).

Past Psychiatric History: patient was diagnosed with schizophrenia in June 2004. He was hospitalized twice in the 2014 due to acute psychotic symptoms and aggression. He had adequate trials of olanzapine, haloperidol, and risperidone in the past, with only minor improvement in symptoms, rendering his course as “treatment resistant.”

Social History: patient was born in New York, a product of a full term pregnancy via vaginal delivery, with no complications per mother. He is currently in the 12th grade, special education classes, is unemployed, and is living with mother and two sisters. As per Individualized Educational Program (1/13/14), there was classification of speech and language impairment, with cognitive functioning in the extremely low range (third grade level functioning in all subjects). There is no known history of physical, sexual, or emotional abuse in the past. There is no history of substance or illicit drug use.

## 3. Hospital Course

On the unit, the patient initially displayed disorganized speech and behavior. He was observed showering (multiple times per day) with clothing on, pacing hallways aimlessly, going into other people's bedrooms, and exposing himself to female peers. He displayed minimal social contact with peers and staff. His thought process was tangential, displaying thought blocking, and he would laugh inappropriately. He was restarted on previous discharge medication (quetiapine 200 mg oral (PO) twice a day) on admission but since previous antipsychotic trials were unsuccessful, a clozapine trial was discussed with family. On two separate occasions (9/28/14 and 9/30/14), he was physically aggressive towards his 1 : 1 staff member without a known precipitating event. On 9/30/14, following discussion of risk, benefits, and alternatives, mother gave consent for a clozapine trial and patient was started on 12.5 mg PO daily, with the intention to titrate upwards to reach target range of 300–400 mg PO once a day. At this point in time, EKG showed normal sinus rhythm with normal QT interval. On 10/1/14, patient physically assaulted another staff member and was referred to the Behavioral Support Team (BST) for observation to identify possible triggers or warning signs. Clozapine was titrated (increase of 25 mg every other day) and quetiapine was slowly titrated down and discontinued. Following a week on clozapine (25 mg PO every morning and 100 mg every night), improvement in behavior was noted, as patient was able to engage more appropriately with peers and staff, with no aggressive episodes noted.

**Figure 1:**
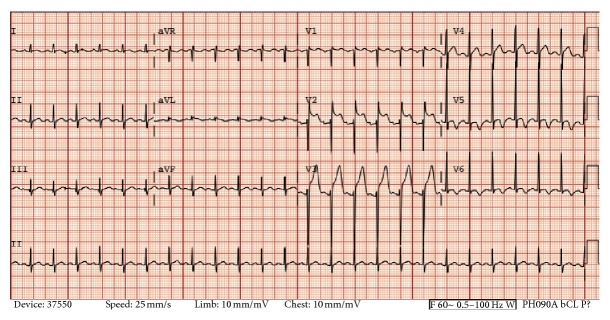
EKG-10/13/14: sinus tachycardia (HR 149), LVH by voltage, and anterior ST elevation (QTc 397 ms).

On the morning of October 12, 2014 (current dose of clozapine 50 mg PO every morning and 100 mg every night), patient appeared irritable and lethargic and reported some gastrointestinal discomfort. During that day, patient was able to eat his meals and attended some unit activities for some time but spent most of the day in his bed (atypical behavior). Vitals done at the time showed a temperature of 97.8°F, pulse of 120 beats/minute, respiration rate of 18 per minute, and blood pressure (BP) of 120/68 mm of Hg. EKG showed sinus tachycardia (pulse 130 beats/minute) and pediatric medical and cardiology attendings were notified. Initially, tachycardia was viewed as a known side effect for clozapine, and medical team advised observation, and the bedtime dose of clozapine was held. On 10/13/14 (day 13 of clozapine trial), patient refused breakfast stating that his “stomach hurts” and labs and vitals were completed (of significance, pulse 120 beats/minute, troponin 11.089 *μ*g/L, creatine kinase (CK) 418 *μ*g/L, creatinine kinase-myocardial b fraction (CK-MB) 31.08 *μ*g/L, and WBC 12.13 cells/mcL). EKG showed sinus tachycardia, left ventricular hypertrophy, and anterior ST elevation (Figure 1).

**Figure 2:**
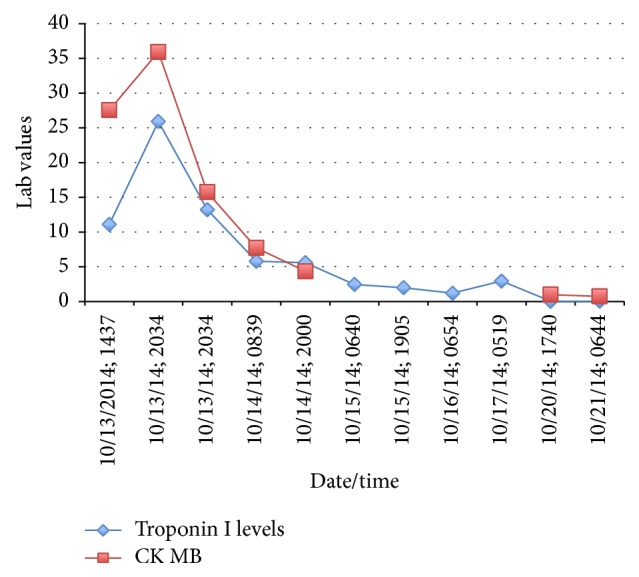
Troponin I (*μ*g/L) and CK MB Trends (*μ*g/L). CK MB levels were not obtained from 10/15/14 to 10/17/15.

The patient was transferred to the pediatric intensive care unit (PICU) for suspected clozapine-induced myocarditis and clozapine was subsequently discontinued on 10/13/14. Echocardiogram on 10/14/14 showed left ventricular ejection fraction of 42.794%, with mild left ventricular dysfunction. He was placed on lorazepam 2 mg PO as needed (PRN) dosing for episodes of agitation/aggression. Subsequently in the PICU, he received PRN medication at least once/day for episodes of aggression (physically assaulting nursing staff). In the PICU, vital signs, troponin, and CK-MB levels were monitored (Figure 2) and he received supportive care with continuous cardiopulmonary monitoring. Repeat echocardiogram showed normal left ventricle size and configuration (ejection fraction within normal range at 66.43%). Upon medical clearance (10/17/14), he was transferred back to psychiatric unit for continuation of psychiatric treatment. He was started on perphenazine 4 mg PO daily and quetiapine 400 mg PO twice daily upon return, with mild improvement in psychosis. Following subsequent two weeks of inpatient treatment, he was discharged, with parental consent, for state hospitalization for continuity of care.

## 4. Discussion

The patient developed clozapine-induced myocarditis on the thirteenth day of clozapine commencement. Despite the fact that the patient was routinely observed in an inpatient setting, the clinical signs of tachycardia, lethargy, and vague gastrointestinal distress yielded low initial suspicion of myocarditis. Transient tachycardia and/or hypotension are often considered benign, common findings with the initiation of clozapine treatment and can be usually managed with supportive care [6]. However, high clinical suspicion for myocarditis must still be maintained.

To date, there have been limited documented cases in the pediatric population, most probably due to its limited usage. A retrospective chart review of 36 youth patients (age 9–21 years) showed that although 66% had at least one criterion indicative of pericarditis, myocarditis, or cardiomyopathy, all abnormalities were found to be nonspecific findings that did not progress into cardiovascular complications [7].

Although there are no specific monitoring guidelines for clozapine-induced myocarditis in the Unities States [8], other countries have proposed recommendations based on adult cases. In Australia, Ronaldson et al. [5] developed an evidence-based monitoring tool based on 75 cases (ages 21–73; mean 38 +/− 12) and 94 controls (ages 19–73; mean 34 +/− 11) for routine monitoring up to day 28. Per algorithm, echocardiography, troponin I or T, and CRP should be completed at baseline, with routine vitals every other day, and troponin and CRP should be repeated on days 7, 14, 21, and 28. If troponin is >2 times the upper limit of the normal (0.03–0.6 *μ*g/L) or C-reactive protein >100 mg/L, clozapine should be discontinued and repeat echocardiography and consult cardiology should be made. If this protocol had been applied to this case, the combination of gastrointestinal distress with tachycardia would prompt team to obtain troponin I and CRP levels (which would be ideally compared to baseline values) immediately, resulting in earlier discovery of the myocarditis.

The establishment and implementation of an evidence-based monitoring protocol to promote early identification of clozapine-induced myocarditis warrant further investigation. Although elevated troponin levels have been proven to be a more reliable predictor of myocardial injury than CK, studies have shown a specificity of 89% and a low sensitivity (34%) in cases of myocarditis [9]. In other words, not all cases of suspected myocarditis had an elevated troponin, and not all elevated troponin levels are diagnostic for myocarditis. A monitoring guideline may prove to be beneficial but further research is needed to assess whether routine measurements can lead to early detection of myocarditis and prevent discontinuation of clozapine for whom it may prove to be of great benefit.

In addition to laboratory data, clinical symptoms should not be overlooked. Particularly in this case report, the patient may have been experiencing prodromal symptoms (i.e., gastrointestinal, respiratory, and urinary) days prior to myocarditis discovery. However, since there is no established protocol to screen for these symptoms, in combination with patient's underlying intellectual disability, time to diagnosis may have been potentially delayed. It may be that a future evidence-based monitoring protocol includes a combination of clinical symptoms as well as objective data (labs, vitals, etc.), to increase likelihood of early detection of clozapine-induced myocarditis.

All health care professionals need to maintain a high index of suspicion for this potentially fatal cardiovascular complication. This case reinforces the need to establish evidence-based monitoring guidelines for clozapine usage, particularly in the pediatric population, where the clinical presentation may be atypical or the potential diagnosis may be overlooked.
